# Talent Selection in Portuguese National Futsal Teams

**DOI:** 10.5114/jhk/199379

**Published:** 2025-10-01

**Authors:** Diogo Mendes, Diogo V. Martinho, Bruno Travassos, Élvio R. Gouveia, Nestor Ordoñez Saavedra, Hugo Sarmento

**Affiliations:** 1 Faculty of Sport Sciences and Physical Education, University of Coimbra, Coimbra, Portugal.; 2 CIPER, FCDEFUC, University of Coimbra, Coimbra, Portugal.; 3 Laboratory of Robotics and Engineering Systems, Interactive Technologies Institute, Funchal, Portugal.; 4 Research Center in Sports Sciences, Health Sciences and Human Development (CIDESD), Department of Sport Sciences, Universidade da Beira Interior, Covilhã, Portugal.; 5 Portugal Football School, Portuguese Football Federation, Portugal.; 6 Department of Physical Education and Sport, University of Madeira, Funchal, Portugal.; 7 Faculty of Health Sciences, Sports Science Program, University of Applied and Environmental Sciences, Bogota, Colombia.

**Keywords:** elite players, indoor soccer, long-term athlete development, talent promotion

## Abstract

This study investigated the talent selection processes implemented in youth and adult national futsal teams. The initial database provided by the Portuguese Football Federation included 668 male futsal players, and statistical analysis was conducted with varying sample sizes. Binary logistic regressions were performed to determine the influence of futsal participation on the likelihood of achieving international status at the adult level. Subsequently, the age of first registration was plotted against the national team in which the first international appearance was recorded. Finally, the selection, re-selection, and de-selection of players from the under-17 youth national team to the adult team were described using frequencies and percentages. For the total sample, the results indicated that an earlier age at first registration, participation in the 1^st^ Division, involvement in foreign competitions, and engagement with multiple clubs increased the likelihood of being selected for the international team. The analysis also considered the year in which youth national teams were developed. Before 1999, players demonstrated earlier registration ages; however, the timing of starting futsal practice became less relevant to attaining international adult status after 1999. Furthermore, a negligible percentage of youth national team players progressed to the senior national team, highlighting the collectivist approach employed by futsal national coaches. The findings of this study suggest that the transition of talent from youth to adult national teams warrants further investigation. Coaches should develop strategies to promote the development of youth talent as they progress towards adult-level status.

## Introduction

The number of participants in competitive futsal has increased in recent years (UEFA, 2023). According to official data from the Portugal Football Observatory, the number of male players rose from 42,580 in the 2010/2011 season to 49,403 in 2019/2020 (Portugal Football Observatory, 2021). The statistics also showed growth in the number of clubs, competitions, young futsal players, and events involving national youth teams (Portugal Football Observatory, 2021). In fact, the Under-19 European Championships (2022, 2023) and the European and World Championships at the senior level (2021, 2022) reflected the success of the strategic investments made by the Portuguese Football Federation (https://www.fpf.pt/pt/Institucional/Compromissos/Compromissos-2020-2024/As-NossasMetas; accessed on 9 April 2024). This program has enabled national teams to select talented players, contributing to their success in the international competitions. Determining the key factors that contribute to reaching the elite level is crucial for creating an effective youth futsal talent development program (Baker et al., 2020; Horrocks et al., 2016).

The concept of talent in the context of sports refers to athletes who exhibit exceptional qualities at a young age, which are indicative of their potential to reach elite levels in the future (Cobley et al., 2020). Talent selection considers various stages of development, allowing for the identification of players who meet specific performance criteria (Till and Baker, 2020). Two theoretical perspectives are commonly referenced to explain the promotion of younger players to elite levels: individualistic and collective approaches (Güllich and Emrich, 2012). The individualistic approach focuses on nurturing players on an individual basis to promote long-term athletic development. In contrast, the collectivist approach emphasizes the continuous selection and de-selection of players across different age groups (Güllich and Emrich, 2012). This collectivist model is evident in European football academies, where elite players emerge through systematic selection and de-selection processes across age groups (Ford et al., 2022; Güllich, 2014). However, existing studies that investigate the success of talent programs are primarily limited to football samples. Moreover, these analyses often focus on the percentages of players selected and de-selected from a young age until they reach the professional level. A comprehensive understanding of the variables that explain the attainment of highly competitive standards from a young age necessitates longitudinal or retrospective study designs (Albaladejo Saura et al., 2023; Johnston et al., 2018). Given the popularity and success of Portugal in futsal at the international level (Sarmento et al., 2015; Travassos et al., 2023), there is a noticeable gap in understanding the talent program developed by the Portuguese Football Federation aimed to potentiate elite players.

A recent review summarized the literature on talent identification in futsal players (Mendes et al., 2022). That study identified three key constraints—task, athlete, and environment—as determinants of talent selection in futsal (Davis et al., 2013; Sarmento et al., 2018). In terms of task constraints, the specificity and amount of practice in futsal were found to be crucial in distinguishing between elite and non-elite players (Mendes et al., 2022). However, this conclusion was based solely on a single study utilizing self-reported data (Serrano et al., 2013). The review by Mendes et al. (2022) also highlighted that most research in the field was based on cross-sectional study designs and unidimensional perspectives (Duncan et al., 2018; Galy et al., 2015; Lago-Fuentes et al., 2020). Indeed, there is a need for new research in the area of talent identification in futsal that employs more robust study designs (Méndez-Dominguez et al., 2022).

The retrospective analysis of the extensive database of players registered with the Portuguese Football Federation who have reached national team status is essential for enhancing youth development programs based on scientific evidence. Considering the investment and strategic planning by the Portuguese Football Federation in futsal, along with the success of national squads, this study aimed to: (1) investigate the key variables that distinguished players who attained the senior national team (i.e., international AA) from those who did not; and (2) describe the Portuguese talent program in futsal by examining the turnover of players within the national teams.

## Methods

### Ethical Approval and Participants

The study was approved by the Ethics Committee of the Faculty of Sport Sciences and Physical Education, University of Coimbra (approval code: CE/FCDEF-UC/00842021; approval date: 25 March 2022). The database provided by the Portuguese Football Federation included 668 male futsal players registered between 1970 and 2023. Players were born between 1957 and 2006. To complete the cases of missing data, two websites were consulted: https://www.fpf.pt/pt/ and https://www.zerozero.pt/.

### Variables

The following variables were considered in the analysis: age at first registration (the official date of starting futsal competition), total number of seasons played in futsal, the number of seasons as a youth player, the number of seasons competing in the 1^st^ Division, the number of seasons in Secondary Divisions, the number of seasons competing in foreign countries, and the number of clubs represented.

### Statistical Analysis

Binary logistic regressions were conducted to determine the influence of variables related to futsal participation—age at first registration, total number of seasons played in futsal, the number of seasons as a youth player, the number of seasons competing in the 1^st^ Division, the number of seasons in Secondary Divisions, the number of seasons competing in foreign countries, and the number of clubs represented—on the probability of attaining a position in the senior national team (international AA). From the database provided by the Portuguese Football Federation (N = 668), 129 participants were excluded, as they were not yet 17 years old. This age corresponds to the youngest player ever to compete for the Portuguese national senior futsal team. Therefore, it was established as the cutoff criterion. Binary logistic regressions were performed for the entire sample (N = 539), considering two different time periods: players born before 1999, and players born in or after 1999. The year 1999 marked a significant structural change in Portuguese futsal with the development of youth national teams. Consequently, there were 296 players born before 1999 and 243 players born in or after 1999.

The cross-tabulation of the number of international appearances and age at first registration included futsal players classified as international (i.e., those who participated in official games for the youth or adult national teams) (N = 415). The analysis was divided based on the emergence of the under-21, under-19, under-17, and under-15 youth national teams, which occurred with players born after 1999. Prior to 1999, only three official national squads were recognized: senior, under-23, and under-19. A total of 239 international players were registered before 1999, while 176 international players were recorded after this year.

The frequencies and percentages of selected, re-selected, and deselected players included international players born after 1999 (N = 176). Due to a limited number of under-15 players, fourteen individuals were excluded from the analysis. As a result, the final sample comprised 162 international players. The analyses were conducted using SPSS version 27.0 (SPSS Inc., IBM Company, N.Y., USA) and GraphPad Prism (GraphPad Software, San Diego, California, USA, www.graphpad.com).

## Results

**Figure 1:**
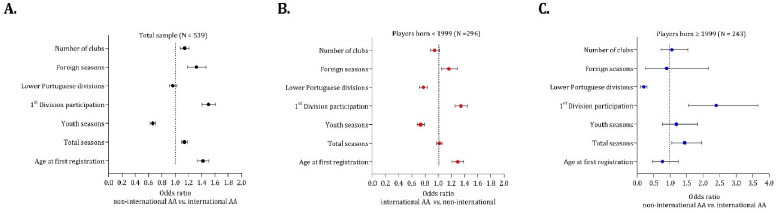
The odds ratios for participation at the adult international level considering three distinct groups: the entire sample (Panel A), players born before 1999 (Panel B), and players born after 1999 (Panel C). International AA (international adult level)

For the entire sample (N = 539), the results indicate that several factors significantly increased the odds ratio (OR) of becoming an international senior player. Specifically, the age at first registration (OR = 1.419, 95% CI: 1.333 to 1.510), total seasons played (OR = 1.139, 95% CI: 1.098 to 1.181), the number of seasons in the 1^st^ Portuguese Division (OR = 1.504, 95% CI: 1.406 to 1.608), foreign seasons (OR = 1.320, 95% CI: 1.188 to 1.465), and the number of clubs represented (OR = 1.143, 95% CI: 1.077 to 1.213) contributed to a higher probability of being selected for the senior national team. An earlier age of registration with the Portuguese Football Federation, an increased number of seasons played in the 1^st^ Division or in leagues outside Portugal, and registration with multiple clubs all contributed to higher likelihood of internationalization in the senior team (Figure 1, panel A).

A similar analysis was conducted considering the year of appearance of youth national teams (1999). Before 1999, the results indicated that a later age of registration in futsal (OR = 1.298; 95% CI: 1.212 to 1.390), a greater number of seasons in the 1^st^ Division (OR = 1.345; 95% CI: 1.256 to 1.440), and participation in foreign clubs (OR = 1.162; 95% CI: 1.048 to 1.288) were associated with a higher chance of becoming an international AA player (Figure 1, panel B). After 1999, the results indicated that the number of seasons played in futsal (OR = 1.436; 95% CI: 1.049 to 1.965) and also the number of seasons in the 1^st^ Division (OR = 2.399; 95% CI: 1.571 to 3.662) were predictive of participation at the international adult level (Figure 1, panel C).

**Figure 2:**
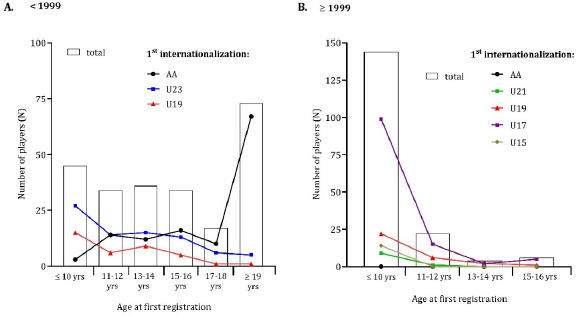
Age of first registration plotted against the first internationalization (number of players). AA (international adult level); U23 (Under-23); U21 (Under-21); U19 (Under-19); U17 (Under-17); U15 (Under-15)

Before 1999, most international players were registered for the first time when they were 19 years old or older (31%). There was a general dispersion in the frequencies of internationalization among players aged 10 to 15–16 years. Notably, players who achieved their first international appearances in under-19 and under-23 teams typically began practicing futsal at the age of 10 or younger, with 33% and 60% starting when they were ≤ 10 years old, respectively. However, 67 players (92%) who achieved their first international appearance in the national senior team started practicing futsal at the age of 19 or older (Figure 2, panel A). Conversely, after 1999, 144 international players were registered for the first time at the age of 10 or younger (82%). No players from this group achieved the adult national team (Figure 2, panel B). Table 1 presents the cross-tabulations of international players by age at the time of first registration and their first internationalization, before and after 1999.

**Table 1:** Cross-tabulations of players by age at first registration and first internationalization in two different periods.

Age at first registration	< 1999					≥ 1999				
	First internationalization					First internationalization				
	AA	U23	U19	Total		U21	U19	U17	U15	Total
≤ 10 yrs	3 (7%)	27 (60%)	15 (33%)	45 (19%)		9 (6%)	22 (26%)	99 (69%)	14 (9%)	144 (82%)
11–12 yrs	14 (41%)	14 (41%)	6 (18%)	34 (14%)		1 (1%)	6 (27%)	15 (68%)	0	22 (13%)
13–14 yrs	12 (33%)	15 (42%)	9 (25%)	36 (15%)		0	2 (50%)	2 (50%)	0	4 (2%)
15–16 yrs	16 (47%)	13 (38%)	5 (15%)	34 (14%)		0	1 (17%)	5 (83%)	0	6 (3%)
17–18 yrs	10 (59%)	6 (35%)	1 (1%)	17 (7%)						
≥ 19 yrs	67 (92%)	5 (7%)	1 (1%)	73 (31%)						
**TOTAL**	239					176				

U15 (Under-15), U17 (Under-17), U19 (Under-19), U21 (Under-21), U23 (Under-23), AA (international adult level

Table 2 summarizes the percentage of selection, reselection, and de-selection from the under-17 to the senior national team. A total of seven players (approximately 4%) progressed from the under-17 team to the senior national team, while only one player advanced from the under-19 team to the senior national team. More than 90% of youth international players never reached the senior national team.

**Table 2:** Selection, deselection and re-selection of futsal players born after 1999.

STATUS	U17	U19	U21	AA
Selected	121 (75%)	31 (19%)	11 (7%)	0 (0%)
Non-selected	40 (25%)	67 (42%)	107 (66%)	154 (95%)
Re-selected	-	64 (40%)	14 (9%)	0 (0%)
Twice re-selected	-	-	30 (19%)	1 (0.6%)
Triple re-selected	-	-	-	7 (4.4%)
**TOTAL**	162			

U17 (Under-17), U19 (Under-19), U21 (Under-21), AA (international adult level)

## Discussion

This study provides new and important insights into the field of talent development in futsal. The total number of seasons played, along with the number of seasons spent in the 1^st^ Division, are crucial for achieving the international adult level. However, the age of first registration within the Portuguese Football Federation and the number of seasons played as youth players do not significantly increase the likelihood of becoming an international player in adulthood. These findings contrast with data from before 1999, which showed that early engagement in futsal was associated with a higher probability of playing for the national adult team. Additionally, the timing of early registration appears to be linked to participation in youth national teams. Since 1999, the lack of youth international players transitioning to the adult national team indicates that the progression from youth to senior levels requires further attention. Moreover, systematic selection and de-selection processes from the under-17 to the adult team were observed, indicating a collectivist approach in the Portuguese futsal system

Two distinct periods were investigated regarding the emergence of youth national teams, which impacted the selection of players for the national adult team. Before 1999, early engagement in futsal was associated with participation in adult national teams; however, an opposing trend emerged after this date. A previous study identified relevant factors that were crucial in distinguishing futsal players by competitive level based on self-reported data (Serrano et al., 2013). In contrast to the findings of the present study, the early practice of futsal was reported as essential for differentiating the twenty-four best national players from those competing at lower levels (N = 270) (Serrano et al., 2013). Differences in sample sizes between groups and the reliance on self-reported data may have biased those results. In the current study, official data provided by the Portuguese Football Federation were utilized. Furthermore, the results align with previous data on German professional soccer players (Güllich, 2014), indicating that the younger players debuted in national youth teams, the lower their probability of attaining the professional championship (Bundesliga). Additionally, the age of entry into youth soccer academies was lower for players competing in the secondary division (13.6 ± 3.9 years) compared to those who played in the Bundesliga (14.3 ± 3.8 years) (Gullich et al., 2014). Those results suggest that the process of talent identification at younger ages does not correlate with achieving international adult levels. Nevertheless, most futsal players who reach the youth international level were registered before the age of 10. While the formation of youth national teams supports the development of futsal players, appropriate environments should be created to facilitate the transition from youth to the adult national team (McGuigan et al., 2024; Morris and Deason, 2020; Van Houten et al., 2019).

The identification of players aged 10 years and younger to create greater opportunities for expanding the talent pool is challenging. Identification is defined as the process of recognizing players from a young age who have the potential to become elite athletes (Till and Baker, 2020). Consequently, identifying talented players requires a multidisciplinary approach and longitudinal data (Bergkamp et al., 2019; Johnston et al., 2018; Sarmento et al., 2018). While a multidimensional approach that tracks youth players over a specific period is documented in soccer (Elferink-Gemser et al., 2007; Figueiredo et al., 2009; Huijgen et al., 2014), no such studies currently exist in futsal. Another critical aspect of talent identification is the prediction of adult performance. A combination of data from 6,233 athletes revealed that elite-performing adult athletes tended to enter talent programs at older ages compared to their lower-performing senior peers (Güllich and Barth, 2024). The process of talent identification raises questions, particularly in sports where research is limited. Several key inquiries should be addressed: (1) Does the identification process consider relevant characteristics at younger ages or should the criteria be based on adult performance? (2) What about athletes who are not labeled as talented? Should they be excluded from the sport or should an environment be fostered to encourage their development?

The repetition of selection and de-selection among players found in this study suggests a collective approach rather than a continuous nurturing process within the national squads. No players from the youth squads have transitioned to the adult international team. Talent development results from the interaction between athletes and constraints related to the individual, the task, and the environment (Araújo and Davids, 2011; Baker et al., 2020). In fact, futsal has been widely recognized by elite soccer coaches as a valid strategy for improving skills and developing multifunctional players (Yiannaki et al., 2018). The talent development process within youth national squads calls for further reflection by the Portuguese Football Federation, particularly regarding how to enhance athletic functionality as players progress from youth to adult international levels. Nonetheless, the development of youth national squads and the investment made at the professional level are two positive examples of promoting talent development. This aligns with the talent development programs implemented in football academies, where the number of matches increases as players advance to adult levels (Ford et al., 2022).

The present study has some limitations that must be considered when interpreting the results. Given the complexity of talent identification, the study is limited to data related to futsal participation. Future research should adopt a longitudinal design that incorporates a combination of physical, physiological, psychological, and socio-cultural variables. Additionally, the establishment of youth national teams necessitates further investigation into the development processes adopted by coaches. Thus, it is essential to create an appropriate environment to nurture youth players during their transition to the adult team.

## Conclusions

The present study provides a critical contribution to research in the field of talent development in futsal. While a collectivist approach has emerged as the strategy used by coaches to select players for the national teams, the need for a long-term continuous nurturing process, beginning when players are registered in youth national squads, requires further research. This approach is essential for developing more players within an appropriate competitive system. Interestingly, the early practice of futsal did not increase the likelihood of becoming an international player in the adult team; however, a significant percentage of players registered in youth national teams were registered before the age of 10. Although the creation of youth teams by the Portuguese Football Federation has proven to be a valuable strategy for promoting the development of futsal players, the transition from youth to adult teams in futsal deserves more attention in future studies.

## References

[ref1] Albaladejo Saura, M., Vaquero-Cristóbal, R., García-Roca, J. A., & Esparza-Ros, F. (2023). What Variables Allow the Differentiation between More and Less Successful Adolescent Volleyball Players?. *Journal of Human Kinetics*, 88, 229–242. 10.5114/jhk/166107 PMC10407323 37559765

[ref2] Araújo, D., & Davids, K. (2011). Talent development: from possessing gifts, to functional environmental interactions. *Talent Development & Excellence*, 3(1), 23–25.

[ref3] Baker, J., Cobley, S., & Schorer, J. (2020). *Talent Identification and Development in Sport* (2^nd^ ed.). Taylor & Francis.

[ref4] Bergkamp, T. L. G., Niessen, A. S. M., den Hartigh, R. J. R., Frencken, W. G. P., & Meijer, R. R. (2019). Methodological Issues in Soccer Talent Identification. *Research Sports Medicine*, 49(9), 1317–1335. 31161402 10.1007/s40279-019-01113-w PMC6684562

[ref5] Cobley, S., Baker, J., & Schorer, J. (2020) Talent identification and development in sport: an introduction to a field of expanding research and practice. In J. Baker, S. Cobley & J. Schorer (Eds.), *Talent Identification and Development in Sport*. Routledge (2^nd^ ed., pp. 1–16). Taylor & Francis.

[ref6] Davids, K., Araújo, D., Vilar, L., Renshaw, I., & Pinder, R. (2013). An ecological dynamics approach to skill acquisition: Implications for development of talent in sport. *Talent Development and Excellence*, 5(1), 21–34.

[ref7] Duncan, S., Oppici, L., Borg, C., Farrow, D., Polman, R., & Serpiello, F. R. (2018). Expertise-related differences in the performance of simple and complex tasks: an event-related potential evaluation of futsal players. *Science and Medicine in Football*, 2(2), 157–162.

[ref8] Elferink-Gemser, M. T., Visscher, C., Lemmink, K. A., & Mulder, T. (2007). Multidimensional performance characteristics and standard of performance in talented youth field hockey players: a longitudinal study. *Journal of Sports Sciences*, 25(4), 481–489. 17365535 10.1080/02640410600719945

[ref9] Figueiredo, A. J., Gonçalves, C. E., Coelho E Silva, M. J., & Malina, R. M. (2009). Characteristics of youth soccer players who drop out, persist or move up. *Journal of Sports Sciences*, 27(9), 883–891. 19629837 10.1080/02640410902946469

[ref10] Ford, P. R., Bordonau, J. L. D., Bonanno, D., Tavares, J., Groenendijk, C., Fink, C., Gualtieri, D., Gregson, W., Varley, M. C., Weston, M., Lolli, L., Platt, D., & Di Salvo, V. (2020). A survey of talent identification and development processes in the youth academies of professional soccer clubs from around the world. *Journal of Sports Sciences*, 38(11–12), 1269–1278. 32378447 10.1080/02640414.2020.1752440

[ref11] Galy, O., Zongo, P., Chamari, K., Chaouachi, A., Michalak, E., Dellal, A., Castagna, C., & Hue, O. (2015). Anthropometric and physiological characteristics of Melanesian futsal players: a first approach to talent identification in Oceania. *Biology of Sport*, 32(2), 135–141. 26060337 10.5604/20831862.1140428 PMC4447759

[ref12] Güllich A. (2014). Selection, de-selection and progression in German football talent promotion. *European Journal of Sport Science*, 14(6), 530–537. 24245783 10.1080/17461391.2013.858371

[ref13] Güllich, A., & Barth, M. (2024). Effects of Early Talent Promotion on Junior and Senior Performance: A Systematic Review and Meta-Analysis. *Sports Medicine*, 54(3), 697–710. 37921913 10.1007/s40279-023-01957-3 PMC10978645

[ref14] Güllich, A., & Emrich, E. (2012). Individualistic and collectivistic approach in athlete support programmes in the German high-performance sport system. *European Journal for Sport and Society*, 9(4), 243–268.

[ref15] Horrocks, D. E., McKenna, J., Whitehead, A., Taylor, P. J., & Morley, A. M. (2016). Qualitative perspectives on how Manchester United Football Club developed and sustained serial winning. *International Journal of Sports Science & Coaching*, 11(4), 467–477.

[ref16] Huijgen, B. C., Elferink-Gemser, M. T., Ali, A., & Visscher, C. (2013). Soccer skill development in talented players. *International Journal of Sports Medicine*, 34(8), 720–726. 23459855 10.1055/s-0032-1323781

[ref17] Johnston, K., Wattie, N., Schorer, J., & Baker, J. (2018). Talent Identification in Sport: A Systematic Review. *Sports Medicine*, 48(1), 97–109. 29082463 10.1007/s40279-017-0803-2

[ref18] Lago-Fuentes, C., Rey, E., Padrón-Cabo, A., Prieto-Troncoso, J., & Garcia-Núñez, J. (2020). The Relative Age Effect in Professional Futsal Players. *Journal of Human Kinetics*, 72, 173–183. 32269658 10.2478/hukin-2019-0105 PMC7126250

[ref19] McGuigan, M., Dello Iacono, A., McRobert, A., Cowan, D., & Unnithan, V. B. (2024). Facilitators and barriers associated with youth player transition to professional first-team football: A key stakeholder perspective. *International Journal of Sports Science & Coaching*, 19(3), 988–998.

[ref20] Mendes, D., Travassos, B., Carmo, J. M., Cardoso, F., Costa, I., & Sarmento, H. (2022). Talent Identification and Development in Male Futsal: A Systematic Review. *International Journal of Environmental Research and Public Health*, 19(17), 10648. 10.3390/ijerph191710648 36078360 PMC9517923

[ref21] Méndez-Dominguez, C., Nakamura, F. Y., & Travassos, B. (2022). Editorial: Futsal Research and Challenges for Sport Development. *Frontiers in Psychology*, 13, 856563. 35386892 10.3389/fpsyg.2022.856563 PMC8978823

[ref22] Morris, R., & Deason, E. (2020). The transition from elite youth to elite adult professional soccer: A summary of current literature and practical applications. In J. Dixon, J. Barker, R. Thelwell & I. Mitchell (Eds.), *The Psychology of Soccer* (pp. 3–18). Routledge.

[ref23] Portugal Football Observatory. (2022). The Evolution of Futsal in Portugal and the Victory in the 2018 European Championship as a Growth Accelerator, *Portuguese Football Federation*.

[ref24] Sarmento, H., Anguera, M. T., Pereira, A., & Araújo, D. (2018). Talent Identification and Development in Male Football: A Systematic Review. *Sports Medicine*, 48(4), 907–931. 29299878 10.1007/s40279-017-0851-7

[ref25] Sarmento, H., Bradley, P., & Travassos, B. (2015). The Transition from Match Analysis to Intervention: Optimising the Coaching Process in Elite Futsal. *International Journal of Performance Analysis in Sport*, 15(2), 471–488.

[ref26] Serrano, J. M. P. R., Santos, S. D. L. D., Sampaio, A. J. E., & Leite, N. M. C. (2013). Sport initiation, early sport involvement and specialization in futsal training in Portugal. *Motriz: Revista de Educação Física*, 19, 99–113.

[ref27] Till, K., & Baker, J. (2020). Challenges and [Possible] Solutions to Optimizing Talent Identification and Development in Sport. *Frontiers in Psychology*, 11, 664. 32351427 10.3389/fpsyg.2020.00664 PMC7174680

[ref28] Travassos, B., Braz, J., Mendes, J. L., Palas, P., Rodrigues, M., Silvério, J., & Brito, J. (2023). The Road to Becoming a World Champion in Futsal. *International Journal of Sports Physiology and Performance*, 18(6), 590–602. 37055023 10.1123/ijspp.2022-0414

[ref29] UEFA Annual Report 2022/2023. Futsal (p.38).

[ref30] Van Houten, J. M., Kraaykamp, G., & Pelzer, B. J. (2019). The transition to adulthood: a game changer!? A longitudinal analysis of the impact of five major life events on sport participation. *European Journal for Sport and Society*, 16(1), 44–63.

[ref31] Yiannaki, C., Carling, C., & Collins, D. (2018). Futsal as a potential talent development modality for soccer–a quantitative assessment of high-level soccer coach and player perceptions. *Science and Medicine in Football*, 2(4), 299–308.

